# A multi-site, multi-year labeled polygon dataset for agricultural land use and land cover mapping in central and northern Benin

**DOI:** 10.1016/j.dib.2026.113272

**Published:** 2026-09-17

**Authors:** Sabi Bruno Bio Nikki Sarè, Raffaele Gaetano, Yvon-Carmen Hountondji, Comlan Hervé Sossou, Laurent Gazull, Gérard Nounagnon Gouwakinnou, Nestor René Ahoyo Adjovi, Simon Madec, Agnès Bégué, Naboua Abdelkader Kouhoundji, Omoto Aurelle Christelle Sedegnan, Mikhaïl Jean De Dieu Dotou Padonou, Coômlan Charles Hounton, Séverin Biaou, Jaurès Kakpo, Samirath Chabi Yoruba, Nicanor Kouton, Bénoît Adinafo Clément Bessan, Biaou Denis Olou, Jean Yarou Koto, Amah Akodewou, Benoit Mertens, Antoine Denis, Bernard Tychon, Jonathan Peereman, Valentine Lebourgeois, Roberto Interdonato, Jean Huchon

**Affiliations:** a CIRAD, UMR TETIS, University of Montpellier, Montpellier, 34090, Hérault, France; b Unité de Recherche en Télédétection Appliquée et Gestion de l’Information Spatialisée, Laboratoire d’Études et de Recherches Forestières (LERF), Faculté d’Agronomie, Université de Parakou, Parakou, 03 BP 125, Borgou, Bénin; c Laboratoire d’Analyse et de Recherche sur la Politique Agricole (LARPA), Institut National des Recherches Agricoles du Bénin (INRAB), Abomey-Calavi, Agonkanmey, 01 BP 884 Cotonou, Bénin; d Laboratoire d’Écologie, de Botanique et de Biologie Végétale, Faculté d’Agronomie, Université de Parakou, Parakou, 03 BP 125, Borgou, Bénin; e Laboratoire Eau Environnement Développement, Département des Sciences et Gestion de l’environnement, Arlon Campus Environnement, Faculté des Sciences, UR SPHERES, Université de Liège, 185 Avenue de Longwy, Arlon, B-6700, Belgium; f Laboratoire d’Hydraulique et de Maîtrise de l’Eau, Université d’Abomey-Calavi, 01 BP 526 Cotonou, Bénin; g UMR Espace-DEV, Institut de Recherche pour le Développement (IRD), Montpellier, France; h INRIA, University of Montpellier, Montpellier, 34090, Hérault, France; i Delegation of the European Union to Benin - EEAS, Cotonou, Bénin

**Keywords:** Labeled dataset, Smallholder agriculture, Sentinel-2, SPOT, West Africa, Northern Benin, LULC mapping, Deep learning

## Abstract

Accurate land use and land cover (LULC) mapping in West African agricultural landscapes is severely constrained by the scarcity of high-quality, geo-referenced reference data. In the central and northern regions of Benin, this limitation is particularly acute due to the high spatial heterogeneity of the landscape, the small size of agricultural plots, the diversity of farming practices, and the logistical challenges of conducting field surveys in remote agro-pastoral areas. This data article addresses this gap by describing a multi-site, multi-annual dataset of geo-referenced labeled polygons for agricultural LULC mapping, produced within the framework of the EU DeSIRA OBSYDYA Project.

The dataset integrates complementary sources of reference information across six pilot sites in central and northern Benin, each covering a 50×50 km${}^{2}$ observation window. Annual field campaigns conducted between 2022 and 2024 provided the primary reference information for annual crops, while a dedicated field campaign documented areas occupied by natural formations. Additional polygons were delineated by direct visual interpretation of SPOT 6/7 pansharpened imagery and, for built-up surfaces, complemented with OpenStreetMap (OSM) data. All polygons were subsequently subjected to expert visual validation and geometric and topological quality control. The nomenclature comprises 13 land cover classes adapted to the local agricultural context, including annual crop types (leguminous/oleaginous crops, cereals, cotton, roots/tubers, and other crops), perennial agroforestry systems (fruit tree crops and other tree crops), natural formations (dense forest, open forest, and shrubby savanna), and non-agricultural classes (built-up surfaces, bare soil, and water).

The dataset contains 33 065 labeled polygons representing 18 189.32 ha of labeled surface across 18 site-year combinations, with a growing spatial coverage over the three years (9 027 polygons in 2022, 9 932 in 2023, and 14 106 in 2024).

The dataset constitutes a solid and unique reference base for central and northern Benin, serving three complementary purposes: the operational production of annual agricultural LULC maps within the OBSYDYA project using the MORINGA processing chain [1]; the design, training, and evaluation of deep learning models for LULC classification; and, more broadly, the benchmarking of supervised classification approaches for the monitoring of agricultural dynamics in Sudanian West Africa.

Field reference data are openly available under a CC-BY licence in the CIRAD GeoDE repository at https://doi.org/10.18167/GEODE/9446 [2].

Specifications Table

**Table:** 

Subject	Computer Science; Earth and Planetary Sciences; Agricultural and Biological Sciences
Specific subject area	Agricultural LULC mapping; Remote sensing; Machine Learning; Deep Learning; Smallholder agriculture
Type of data	Vector (Shapefile .shp) and meta data
Data collection	Annual Crop Types Field Campaigns (October–November 2022–2024), complemented by a dedicated campaign for natural environments and tree crops; field observations collected with Samsung Galaxy Tab S7 FE tablets using QField and SPOT 6/7 pansharpened imagery at 1.5 m as reference. Complementary polygons were derived from expert photo-interpretation and OpenStreetMap for built-up surfaces, followed by expert visual validation and geometric/topological quality control.
Data source location	Six agricultural sites in central and northern Benin, West Africa : Parakou, Ouenou, Bagou, Soaodou, Awanla, Gbanlin.
Data accessibility	Repository: CIRAD GeoDE
	DOI: https://doi.org/10.18167/GEODE/9446
	Licence: CC-BY
Related research article	• Bio Nikki Sarè et al. [3]. MSF-Net: A Multimodal SAR–Optical Fusion Network for Agricultural Land Use Classification in Smallholder Landscapes of Northern Benin. Remote Sensing, 18(15), 2622. https://doi.org/10.3390/rs18152622 • Bio Nikki Sarè et al. [4]. Joint Cloud Removal and Classification of Sentinel-2 Image Time Series for Agricultural Land Cover Mapping in Northern Benin. *IGARSS 2024 – 2024 IEEE International Geoscience and Remote Sensing Symposium*, pp. 4824–4827. 10.1109/IGARSS53475.2024.10641427

## 1 Value of the Data

• The dataset provides a rare, publicly available geo-referenced ground truth for agricultural LULC mapping in central and northern Benin, a context characterized by smallholder farming systems, high landscape fragmentation, a wide diversity of cultivated plants including cereals, cotton, legumes, tubers, market garden crops, and perennial orchards, heterogeneous agricultural practices, frequent intercropping, and a documented scarcity of labeled reference data. It fills a gap that currently limits the development and evaluation of supervised classification approaches for tropical West African agricultural landscapes.
• With three annual campaigns (2022–2024) across six contrasting agro-ecological sites, the dataset enables the training, validation, and benchmarking of machine learning and deep learning classification models under realistic conditions of interannual variability and spatial diversity, including the assessment of model generalization across sites and years.
• The labeled polygons are directly compatible with freely available Sentinel-2 (10 m) and Sentinel-1 imagery (Copernicus/ESA), making the dataset immediately usable for researchers in remote sensing, agricultural monitoring, and food security without requiring access to commercial imagery.
• The nomenclature was designed to reflect the actual land cover classes encountered in the field, including locally relevant categories such as individual annual crop types, fallow vegetation represented within the Shrubby Savannah class, and perennial crops, which are rarely distinguished in global LULC products and are critical for agricultural monitoring in the Sudanian zone of West Africa.
• The dataset has been used operationally to produce annual agricultural LULC maps of the OBSYDYA pilot sites using the MORINGA processing chain [1], and can serve as a benchmark for comparing classification algorithms, multi-sensor fusion strategies, and domain adaptation methods across sites and years.

## 2 Background

Food security is a growing challenge in sub-Saharan Africa, where rapid population growth, agricultural expansion, and increasing climate variability are placing mounting pressure on rainfed smallholder farming systems [5], [6], [7]. In central and northern Benin, agricultural landscapes are undergoing rapid transformations driven by demographic growth, the expansion of commercial crops, and land-use intensification, making accurate and regularly updated land use and land cover (LULC) information essential for agricultural monitoring and evidence-based decision making.

The increasing availability of freely accessible Sentinel-1 and Sentinel-2 imagery, combined with recent advances in machine learning and deep learning, has opened new opportunities for large-scale agricultural LULC mapping. However, the effectiveness of these supervised methods depends critically on the availability of high-quality, representative reference datasets [8]. Despite recent efforts, publicly available reference data remain scarce for West African smallholder systems, limiting the reproducibility and transferability of remote sensing approaches [6], [8].

It is within this context that the OBSYDYA Project^1^ was established to develop an operational satellite-based monitoring system for agricultural landscapes in Benin. To address the identified data gap, we present a multi-site, multi-year labeled polygon dataset covering six study sites and three growing seasons (2022–2024), intended to support supervised classification, model training, validation, and benchmarking. Although other datasets exist for other agricultural systems in the West-African region [9], to the best of our knowledge, no publicly available dataset currently provides multi-site, multi-year, field-referenced agricultural polygons specifically designed for LULC mapping in Benin.

## 3 Data Description

The dataset consists of geo-referenced labeled polygon files, available in vector GIS format, covering six agricultural sites in central and northern Benin across three annual campaigns (2022–2024). It contains a total of 33 065 labeled polygons annotated according to a 13-class local LULC nomenclature (Table 1), representing approximately 18 189.32 ha of labeled surface area. Field reference data are openly available under a CC-BY licence in the CIRAD GeoDE repository at https://doi.org/10.18167/GEODE/9446 [2].

The dataset is organized in the repository by campaign year. For each year, a shapefile containing all labeled polygons across the six sites is provided under the naming convention OBSYDYA_BD_<year>_v2_0_2.shp accompanied by its associated metadata. All vector files use the *WGS 84 / UTM Zone 31N* coordinate system (EPSG:32631). Each polygon is described by the following attributes:

• id: polygon identifier unique within each annual file;
• Site: identifier of the OBSYDYA study site associated with the polygon;
• Source: original annotation source, distinguishing Annual Crop Types Field Campaign, the dedicated Natural Formations Field Campaign, direct Photo-interpretation from VHR Satellite Imagery, and OpenStreetMap-derived annotations;
• Class and CName: integer code and label of the land cover class as recorded in the field;
• Code_L0 and CName_L0: code and label of the Level 0 thematic category (6 classes: Cropland, Tree Crops, Forest, Shrubland, Built-up/Bare Soil, Water);
• Code_L1 and CName_L1: code and label of the Level 1 class (13 classes as defined in Table 1);
• geometry: polygon geometry.

The original annotation provenance is retained in the released dataset through the Source attribute. Four source categories are distinguished: annual field campaigns for annual crops; a dedicated field campaign for natural formations; direct photo-interpretation for visually identifiable surfaces; and OpenStreetMap (OSM) data for built-up surfaces. The proportions of polygons contributed by each source are summarized in Table 2.

**Table 1:** Land cover nomenclature used in the dataset: 13 classes (Level 1) grouped into 6 thematic categories (Level 0).

Level 0 (L0)		Level 1 (L1)		Description
Code	Class name	Code	Class name	

1	Cropland	11	Leguminous / Oleaginous	*Glycine max, Arachis hypogaea*
		12	Cereals	*Zea mays, Sorghum bicolor, Pennisetum glaucum*
		13	Cotton	*Gossypium hirsutum*
		14	Roots / Tubers	*Manihot esculenta, Dioscorea* spp.
		15	Other Crops	Vegetables and other annual crops
2	Tree Crops	21	Fruit Tree Crops	*Anacardium occidentale, Mangifera indica*
		22	Other Tree Crops	*Tectona grandis, Gmelina arborea*
3	Forest	31	Dense Forest	Gallery forests, riparian vegetation, dense woody formations
		32	Open Forest	Open woodland and tree savanna
4	Shrubland	41	Shrubby Savannah	Shrubby and herbaceous fallow; degraded savanna
5	Built-up / Bare Soil	51	Built-up Surfaces	Urban and rural settlements, roads
		52	Bare Soil	Bare, degraded or eroded surfaces
6	Water	61	Water	Rivers, ponds, reservoirs

**Table 2:** Provenance of the labeled polygons by original annotation source.

Original source	Number of polygons	Proportion (%)
Annual Crop Types Field Campaign	26,988	81.6
OpenStreetMap	3183	9.6
Natural Formations Field Campaign	2798	8.5
Photo-interpretation from VHR Satellite Imagery	96	0.3
**Total**	**33065**	**100.0**

Table 3 summarizes the number of labeled polygons and total labeled area per site and year. Reference data are available for all six sites across the three years (2022–2024), although the availability of specific LULC classes varies among site–year combinations, particularly for annual crop classes. The total labeled area increases from 5412.51 ha in 2022 to 5540.77 ha in 2023 and 7236.04 ha in 2024, corresponding to 9027, 9932, and 14,106 labeled polygons, respectively. This increase reflects the progressive expansion of the annual annotation coverage and the resulting increase in the number of labeled areas represented in the dataset.

**Table 3:** Number of labeled polygons and total labeled area (ha) per site and year, computed on the Sentinel-2 pixel grid at 10 m resolution.

Site	2022		2023		2024	
	Polygons	Area (ha)	Polygons	Area (ha)	Polygons	Area (ha)
Awanla	1239	689.09	1577	754.33	3132	1066.66
Bagou	1955	731.34	1409	602.49	3046	1322.58
Gbanlin	1318	1226.78	1924	1350.34	1783	1331.31
Ouenou	1858	1053.79	1575	875.42	2143	1191.45
Parakou	2240	1328.28	2826	1505.91	2761	1581.96
Soaodou	417	383.23	621	452.28	1241	742.08
**Total**	**9027**	**5412.51**	**9932**	**5540.77**	**14106**	**7236.04**

Table 4, Table 5–6 provide a detailed per-class breakdown of polygon counts, labeled areas, and the corresponding number of Sentinel-2 pixels intersecting the labeled polygons on the 10 m processing grid. Fig. 2 shows the spatial distribution of the six sites, and Fig. 1 illustrates examples of labeled polygons overlaid on SPOT 6/7 imagery.

**Table 4:** Number of labeled polygons by LULC class, site, and year. The symbol “–” indicates that no field campaign was conducted and no corresponding annotation data were available for the given site–year combination.

LULC class	Awanla			Bagou			Gbanlin			Ouenou			Parakou			Soaodou		
	2022	2023	2024	2022	2023	2024	2022	2023	2024	2022	2023	2024	2022	2023	2024	2022	2023	2024
Leg./Oleag.	–	49	226	251	178	185	–	359	294	468	305	481	354	430	351	–	56	194
Cereals	–	191	1271	950	733	1925	–	139	211	375	304	609	399	789	815	–	75	457
Cotton	–	10	20	465	235	648	–	22	6	104	64	118	58	96	97	–	13	82
Roots/Tubers	–	76	328	11	0	13	–	63	106	22	28	37	28	71	72	–	10	63
Other Crops	–	8	45	3	1	2	–	20	31	0	0	13	11	32	28	–	0	1
Fruit Tree Crops	513	513	513	47	47	47	718	718	718	372	372	372	369	369	369	100	100	100
Other Tree Crops	198	202	202	55	42	53	183	186	0	150	135	146	93	111	101	12	62	39
Dense Forest	39	39	39	1	1	1	6	6	6	32	32	32	7	7	7	10	10	10
Open Forest	43	43	43	3	3	3	93	93	93	137	137	137	52	52	52	47	47	47
Shrubby Savannah	84	84	83	56	56	56	149	149	149	38	38	38	28	28	28	108	108	108
Built-up Surfaces	315	315	315	78	78	78	135	135	135	131	131	131	802	802	802	123	123	123
Bare Soil	43	43	43	14	14	14	18	18	18	16	16	16	20	20	20	12	12	12
Water	4	4	4	21	21	21	16	16	16	13	13	13	19	19	19	5	5	5
**Total**	**1239**	**1577**	**3132**	**1955**	**1409**	**3046**	**1318**	**1924**	**1783**	**1858**	**1575**	**2143**	**2240**	**2826**	**2761**	**417**	**621**	**1241**

**Table 5:** Labeled area (ha) by LULC class, site, and year, computed from the Sentinel-2 pixel grid at 10 m resolution. The symbol “–” indicates that no field campaign was conducted and no corresponding annotation data were available for the given site–year combination.

(a) Awanla, Bagou, and Gbanlin									
LULC class	Awanla			Bagou			Gbanlin		
	2022	2023	2024	2022	2023	2024	2022	2023	2024
Leg./Oleag.	–	7.40	44.27	52.70	37.93	69.17	–	75.60	123.87
Cereals	–	32.57	262.98	289.10	267.41	797.54	–	30.66	66.14
Cotton	–	1.35	6.04	169.15	80.41	234.62	–	7.91	5.45
Roots/Tubers	–	11.51	49.72	1.44	0.00	3.44	–	9.49	24.58
Other Crops	–	0.95	7.30	0.85	0.57	0.27	–	3.44	9.67
Fruit Tree Crops	240.91	240.91	240.91	32.02	32.02	32.02	639.07	639.07	639.07
Other Tree Crops	73.65	85.12	81.48	26.66	24.73	26.10	125.18	121.64	0.00
Dense Forest	50.47	50.47	50.47	1.54	1.54	1.54	2.14	2.14	2.14
Open Forest	55.52	55.51	55.51	1.91	1.91	1.91	70.43	70.43	70.43
Shrubby Savannah	155.23	155.23	154.67	88.44	88.44	88.44	321.22	321.22	321.22
Built-up Surfaces	76.41	76.41	76.41	15.68	15.68	15.68	43.99	43.99	43.99
Bare Soil	11.96	11.96	11.96	5.85	5.85	5.85	4.50	4.50	4.50
Water	24.94	24.94	24.94	46.00	46.00	46.00	20.25	20.25	20.25

**Total**	**689.09**	**754.33**	**1066.66**	**731.34**	**602.49**	**1322.58**	**1226.78**	**1350.34**	**1331.31**

**(b) Ouenou, Parakou, and Soaodou**									

	**Ouenou**			**Parakou**			**Soaodou**		
**LULC class**	**2022**	**2023**	**2024**	**2022**	**2023**	**2024**	**2022**	**2023**	**2024**

Leg./Oleag.	200.36	84.54	179.33	150.02	164.81	172.58	–	8.22	76.20
Cereals	124.49	87.13	261.66	144.51	290.49	295.98	–	22.06	205.81
Cotton	61.55	23.67	71.66	51.77	48.60	110.64	–	4.32	40.39
Roots/Tubers	5.41	6.60	6.84	3.86	11.83	19.38	–	0.96	15.65
Other Crops	0.00	0.00	1.97	2.21	8.15	4.15	–	0.00	0.07
Fruit Tree Crops	337.13	337.13	337.13	431.18	431.18	431.18	78.55	78.55	78.55
Other Tree Crops	84.36	95.86	92.37	67.32	73.44	70.64	3.17	36.59	23.90
Dense Forest	53.83	53.83	53.83	16.06	16.06	16.06	2.10	2.10	2.10
Open Forest	117.50	117.50	117.50	42.20	42.20	42.20	37.63	37.63	37.63
Shrubby Savannah	25.04	25.04	25.04	48.99	48.99	48.99	222.40	222.47	222.40
Built-up Surfaces	31.72	31.72	31.72	221.39	221.39	221.39	28.54	28.54	28.54
Bare Soil	4.40	4.40	4.40	80.48	80.48	80.48	1.53	1.53	1.53
Water	8.00	8.00	8.00	68.29	68.29	68.29	9.31	9.31	9.31

**Total**	**1053.79**	**875.42**	**1191.45**	**1328.28**	**1505.91**	**1581.96**	**383.23**	**452.28**	**742.08**

**Table 6:** Number of Sentinel-2 pixels intersecting the labeled polygons by LULC class, site, and year on the 10 m processing grid. The symbol “–” indicates that no field campaign was conducted and no corresponding annotation data were available for the given site–year combination.

(a) Awanla, Bagou, and Gbanlin									
LULC class	Awanla			Bagou			Gbanlin		
	2022	2023	2024	2022	2023	2024	2022	2023	2024
Leg./Oleag.	–	740	4427	5270	3793	6917	–	7560	12,387
Cereals	–	3257	26,298	28,910	26,741	79,754	–	3066	6614
Cotton	–	135	604	16,915	8041	23,462	–	791	545
Roots/Tubers	–	1151	4972	144	0	344	–	949	2458
Other Crops	–	95	730	85	57	27	–	344	967
Fruit Tree Crops	24,091	24,091	24,091	3202	3202	3202	63,907	63,907	63,907
Other Tree Crops	7365	8512	8148	2666	2473	2610	12,518	12,164	0
Dense Forest	5047	5047	5047	154	154	154	214	214	214
Open Forest	5552	5551	5551	191	191	191	7043	7043	7043
Shrubby Savannah	15,523	15,523	15,467	8844	8844	8844	32,122	32,122	32,122
Built-up Surfaces	7641	7641	7641	1568	1568	1568	4399	4399	4399
Bare Soil	1196	1196	1196	585	585	585	450	450	450
Water	2494	2494	2494	4600	4600	4600	2025	2025	2025

**Total**	**68909**	**75433**	**106666**	**73134**	**60249**	**132258**	**122678**	**135034**	**133131**

**(b) Ouenou, Parakou, and Soaodou**									

	**Ouenou**			**Parakou**			**Soaodou**		
**LULC class**	**2022**	**2023**	**2024**	**2022**	**2023**	**2024**	**2022**	**2023**	**2024**

Leg./Oleag.	20,036	8454	17,933	15,002	16,481	17,258	–	822	7620
Cereals	12,449	8713	26,166	14,451	29,049	29,598	–	2206	20,581
Cotton	6155	2367	7166	5177	4860	11,064	–	432	4039
Roots/Tubers	541	660	684	386	1183	1938	–	96	1565
Other Crops	0	0	197	221	815	415	–	0	7
Fruit Tree Crops	33,713	33,713	33,713	43,118	43,118	43,118	7855	7855	7855
Other Tree Crops	8436	9586	9237	6732	7344	7064	317	3659	2390
Dense Forest	5383	5383	5383	1606	1606	1606	210	210	210
Open Forest	11,750	11,750	11,750	4220	4220	4220	3763	3763	3763
Shrubby Savannah	2504	2504	2504	4899	4899	4899	22,240	22,247	22,240
Built-up Surfaces	3172	3172	3172	22,139	22,139	22,139	2854	2854	2854
Bare Soil	440	440	440	8048	8048	8048	153	153	153
Water	800	800	800	6829	6829	6829	931	931	931

**Total**	**105379**	**87542**	**119145**	**132828**	**150591**	**158196**	**38323**	**45228**	**74208**

**Fig. 2:**
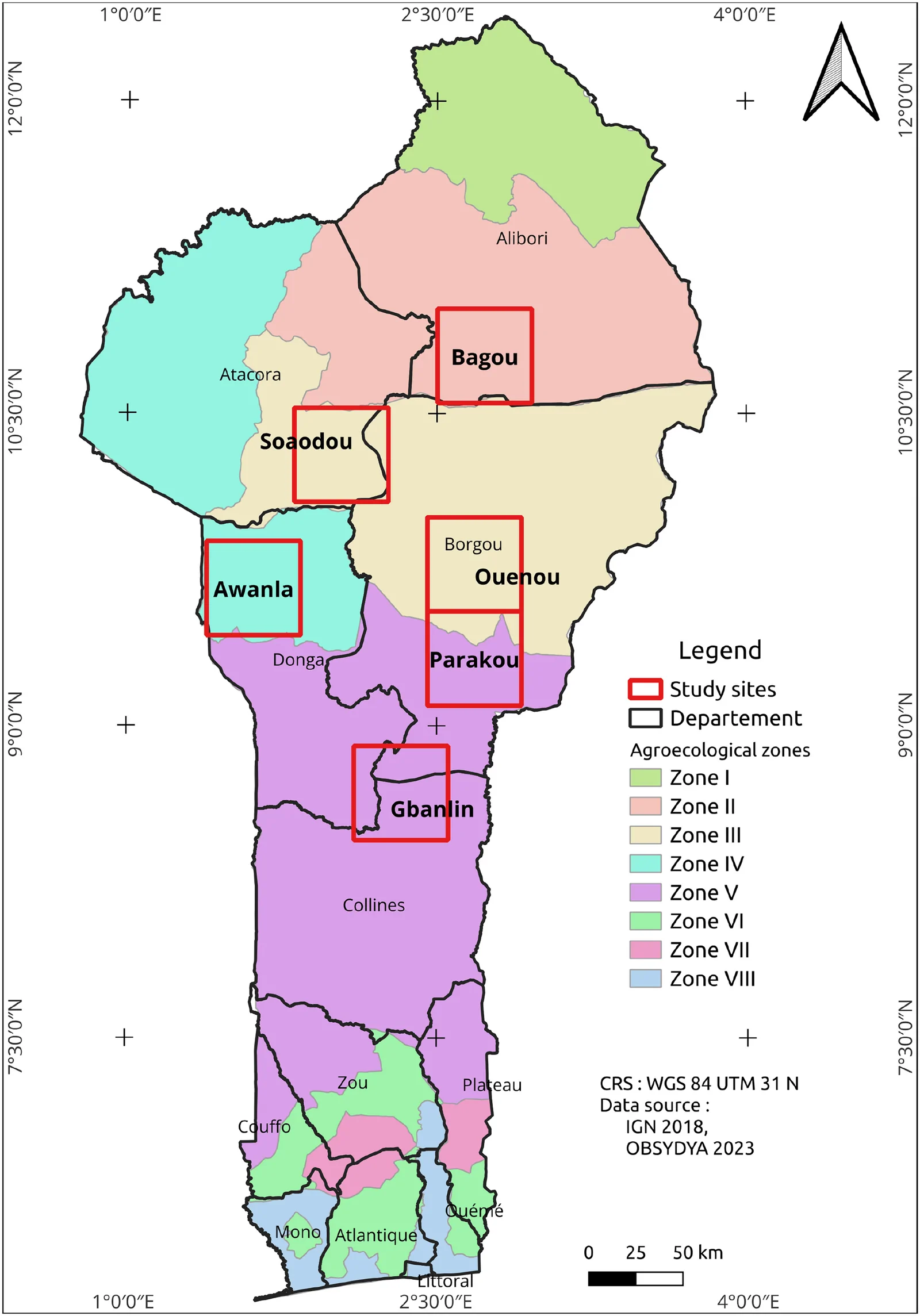
Spatial distribution of the six OBSYDYA study sites in central and northern Benin.

**Fig. 1:**
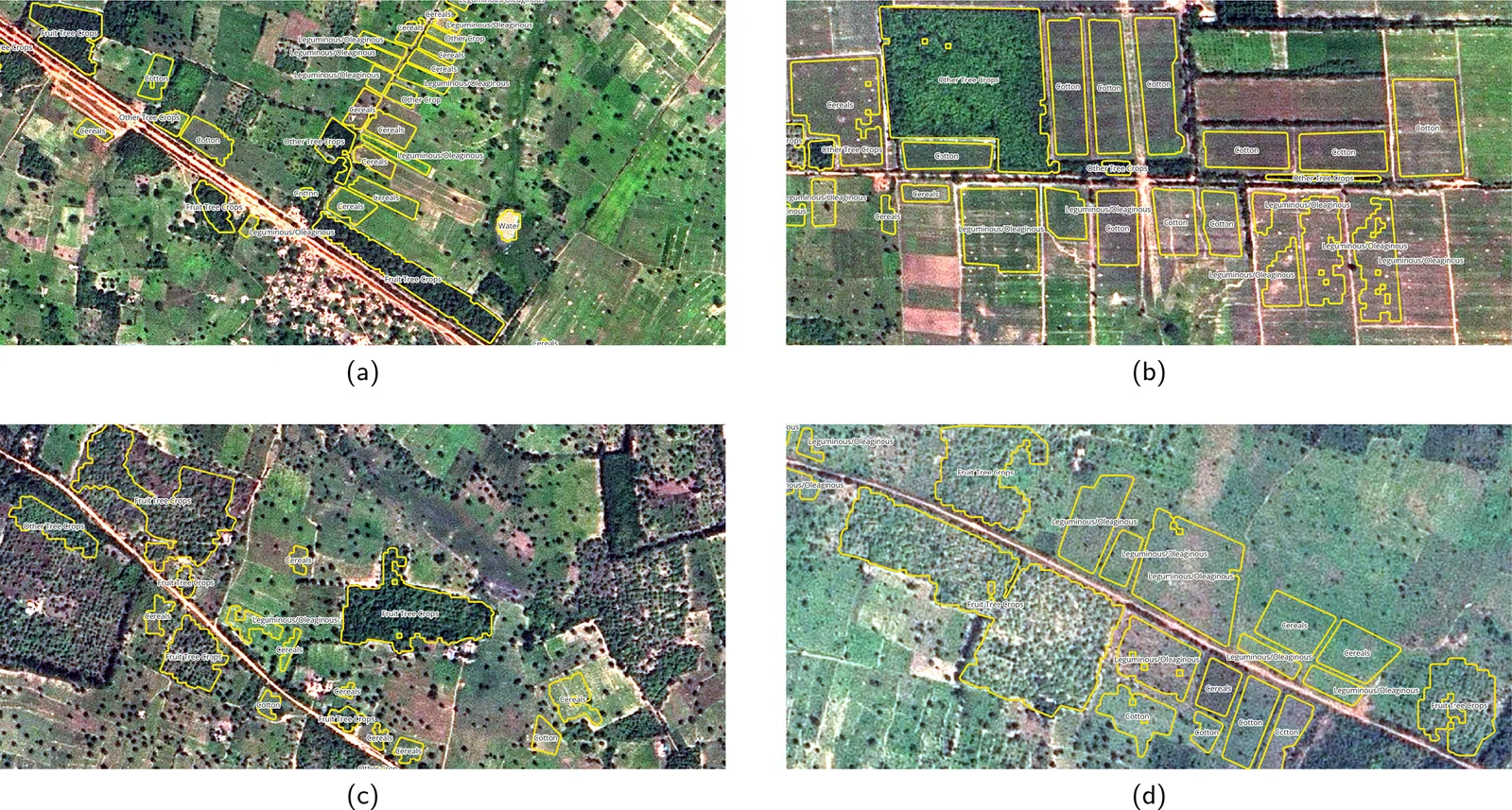
Extracts from the labeled polygon dataset overlaid on SPOT 6/7 pansharpened imagery (1.5 m), illustrating the diversity and spatial complexity of the annotated land cover mosaic. Each polygon represents a labeled homogeneous land-cover unit derived from field observations, direct photo-interpretation, or ancillary OSM data, followed by expert visual validation.

## 4 Experimental Design, Materials and Methods

### 4.1 Study area

The dataset covers six agricultural sites located in central and northern Benin (Fig. 2), each corresponding to a 50×50 km${}^{2}$ observation window centered on a research and development site of Benin’s National Agricultural Research Institute (INRAB^2^), monitored annually within the OBSYDYA project. The region is characterized by a unimodal Sudanese tropical climate, with a dry season from October to April and a rainy season from May to September during which all agricultural activities take place. Annual rainfall ranges from 900 to 1 200 mm and temperatures remain between 25 and 32×C. The landscape is dominated by smallholder farming mosaics, with plot sizes typically between 0.25 and 1 ha, organized in a mosaic of cultivated fields, fallow vegetation within shrubby savanna, woody savannas, and agroforestry plantations.

The six sites encompass diverse agricultural landscapes shaped by contrasting climatic, edaphic, and socio-economic conditions (Table 7). **Awanla** (Zone IV, Atacora West) is characterized by cereal and tuber-based systems on shallow soils. **Bagou** (Zone II, Northern Cotton Basin) represents cotton and cereal-based systems with intensive cattle farming. **Soaodou** and **Ouenou** (Zone III, South Borgou) both reflect typical agropastoral dynamics: Soaodou combines subsistence crops with cotton and cashew production, while Ouenou features residual forest cover within a diversifying landscape (soybean, cotton, food crops, cashew). **Parakou** (Zone V, peri-urban) combines market gardening with cereals and cash crops. Finally, **Gbanlin** (Zone V, Central Cotton Basin) presents the highest crop diversity, with cereals, legumes, cotton, tubers, and perennial plantations.

**Table 7:** Agro-ecological characteristics of the six study sites.

Site	Zone	Dominant systems
Awanla	IV – Atacora West	Cereals, root crops (shallow soils)
Bagou	II – Northern cotton basin	Cotton, cereals, cattle farming
Soaodou	III – South Borgou	Subsistence food crops, cotton, cashew
Ouenou	III – South Borgou	Soybean, cotton, food crops, cashew; residual forest patches
Parakou	V – Central basin (peri-urban)	Market gardening, cereals, cash crops
Gbanlin	V – Central cotton basin	Cereals, legumes, cotton, tubers, perennials

### 4.2 Reference imagery: SPOT 6/7 pansharpened products

SPOT 6/7 pansharpened images at 1.5 m spatial resolution were acquired for each site a few days before each annual field campaign to ensure temporal proximity with field conditions. These products were generated by fusing the 1.5 m panchromatic band with the four 6 m multispectral bands (blue, green, red, near-infrared) using the Bayesian data fusion method [10] implemented in Orfeo ToolBox [11]. SPOT 6/7 images were used exclusively as a basemap for field survey support and polygon delineation, and are not distributed as part of this dataset.

### 4.3 Field survey protocol

Field campaigns were conducted annually between October and November, corresponding to the late growing season when the dominant annual crops (cereals, legumes, cotton, and root crops) have reached advanced phenological stages and can be reliably identified in the field. Surveys followed pre-defined transects established prior to each campaign to ensure systematic and representative spatial coverage of all land cover types present at each site, with particular attention to classes that are frequently confused due to similar spectral or structural characteristics (e.g., fallow vs. degraded natural vegetation, cashew vs. mixed agroforestry, young cotton vs. other annual crops).

Field observations were collected using Samsung Galaxy Tab S7 FE tablets running QField^3^, with site-specific projects incorporating SPOT 6/7 pansharpened imagery at 1.5 m as the reference basemap. The integrated GPS was used primarily to position and navigate the field teams along predefined transects, rather than as a survey-grade GNSS instrument. The collection protocol allow points to be recorded as GPS offsets by identifying the extent and type of nearby surfaces on the VHR satellite image base map; therefore, no survey-level positional accuracy is assigned to the recorded points. Four field teams, each composed of four members and supervised by a team leader, participated in the campaigns. The teams brought together complementary expertise in agronomy, geomatics, and computer science. For each observation, the geographic coordinates and land-cover class according to the local nomenclature (Table 1) were recorded, together with descriptive information when relevant.

### 4.4 Polygon delineation

Following the field campaigns, the recorded observation points were used as spatial references for the delineation of homogeneous land-cover units. For each observation point, the corresponding land-cover class was first reviewed and the surrounding area was interpreted on the SPOT 6/7 pansharpened imagery in QGIS. A polygon was then delineated around the most spatially homogeneous and visually identifiable area corresponding to the observed class. Homogeneity was assessed from the visual consistency of the land-cover unit in terms of texture, spectral appearance, and vegetation structure. Several rules were applied during polygon delineation to ensure thematic and geometric consistency. A polygon was not allowed to intersect a road or to contain distinct land-cover classes associated with different ground observations. Polygons were also required to be non-overlapping. When several ground observations of the same class were located close to each other within a single visually homogeneous area, they could be represented by a single polygon. Conversely, observations of the same class were not aggregated when they were separated by visually heterogeneous areas. Ground observations located very close to one another but associated with different land-cover classes were excluded from polygonization when their spatial configuration would have resulted in very small or mixed polygons.

The resulting polygons therefore represent spatially homogeneous labeled land-cover units rather than direct buffers around individual field observation points. This approach combines the thematic information collected in the field with the finer spatial detail available from the SPOT 6/7 imagery and provides polygon boundaries suitable for subsequent spatial analysis and supervised classification.

### 4.5 Complementary annotation and temporal updating

The reference database integrates several complementary sources of annotation according to the type and temporal stability of the land-cover classes. Annual field campaigns constitute the primary source for annual crops, whose spatial distribution and land-cover status may change substantially between agricultural seasons. Consequently, new field observations were collected during each annual campaign to document the current distribution of these classes. Natural vegetation formations were supported by a dedicated field campaign. Because these land-cover types, along with tree crops, are generally more spatially persistent than annual crops, their reference polygons can be retained across successive campaigns and re-evaluated annually against the available imagery and field information. This applies in particular to natural formations and perennial plantations such as teak and cashew. Polygons were retained only when their thematic identity and spatial homogeneity remained sufficiently consistent over time. Additional polygons were generated through direct visual interpretation of the SPOT 6/7 imagery, particularly for land-cover features that can be identified reliably from very-high-resolution imagery, such as bare-soil surfaces and selected built-up and water features. Built-up areas were also complemented using OpenStreetMap (OSM) data.

Regardless of their original source, all candidate polygons were subjected to expert visual interpretation using the SPOT 6/7 pansharpened imagery. Thus, the annotation source records the provenance of the original reference information, whereas the final polygon geometry and thematic label result from the common delineation and validation procedure described in this section. The original annotation source is explicitly recorded in the Source attribute of the released dataset.

### 4.6 Quality control and thematic validation

Quality control was conducted after polygon delineation and included geometric, topological, and thematic checks. The geometric review focused on the spatial consistency of polygon boundaries with the interpreted land-cover units and on the absence of visible digitizing artefacts. Particular attention was given to the delineation rules described above, including the exclusion of polygons intersecting roads, polygons containing distinct land-cover classes, and overlapping or spatially ambiguous polygons. A systematic topological check was subsequently performed in QGIS using the Topology Checker. The final database was checked for multipart geometries, internal holes, duplicate features, invalid geometries, and polygon overlaps. These checks ensured that each retained feature corresponded to a valid and spatially coherent polygon suitable for GIS-based analysis.

In addition, thematic validation was performed through expert visual interpretation of the SPOT 6/7 imagery, with the original field observations and available ancillary information used as complementary evidence. For annual crops, non-representative portions of an observed area, such as portions affected by tree cover, were removed when they could be identified from the imagery. For natural environments, polygons showing strong internal heterogeneity in vegetation density or structure were excluded rather than assigning a potentially ambiguous label to the entire area. These procedures were intended to retain only polygons for which the assigned land-cover class could be interpreted with sufficient confidence and spatial homogeneity.

The resulting database therefore combines source-specific reference information with a common expert validation and quality-control framework. The numbers of individual polygons corrected, removed, or merged during the original production process were not systematically recorded; consequently, no retrospective quantitative estimate of these operations is reported.

### 4.7 Annual LULC map production using the MORINGA processing chain

The labeled polygon dataset has also been used as reference data for the operational production of annual agricultural LULC maps within the OBSYDYA project, using the MORINGA (*Automatic object-based land cover classification of tropical agrosystems using multi-sensor satellite imagery*) processing chain [1]. MORINGA follows an object-based supervised classification approach that combines very-high-resolution SPOT 6/7 imagery for object delineation with multi-temporal Sentinel-2 observations and a Random Forest classifier. In the present application, the Sentinel-2 time series was generated from atmospherically corrected THEIA products [12], using ten spectral bands resampled to a common 10 m spatial resolution and temporally gap-filled on a 15-day interval [13]. The reference polygons were intersected with the image objects to generate training and validation samples, and a stratified five-fold cross-validation grouped by polygon was used to avoid spatial leakage. The resulting maps were produced at 1.5 m spatial resolution according to the Level-1 nomenclature of the dataset.

The MORINGA workflow is summarized in Fig. 3. Its use with the present dataset illustrates how the labeled polygons can support the transition from field-referenced observations to spatially continuous annual LULC products over the 50×50 km${}^{2}$ observation windows. The processing chain has previously been applied to several tropical agricultural landscapes [14], [15], [16], and its source code and a ready-to-use Docker image are openly available at https://forge.inrae.fr/raffaele.gaetano/moringa.

**Fig. 3:**
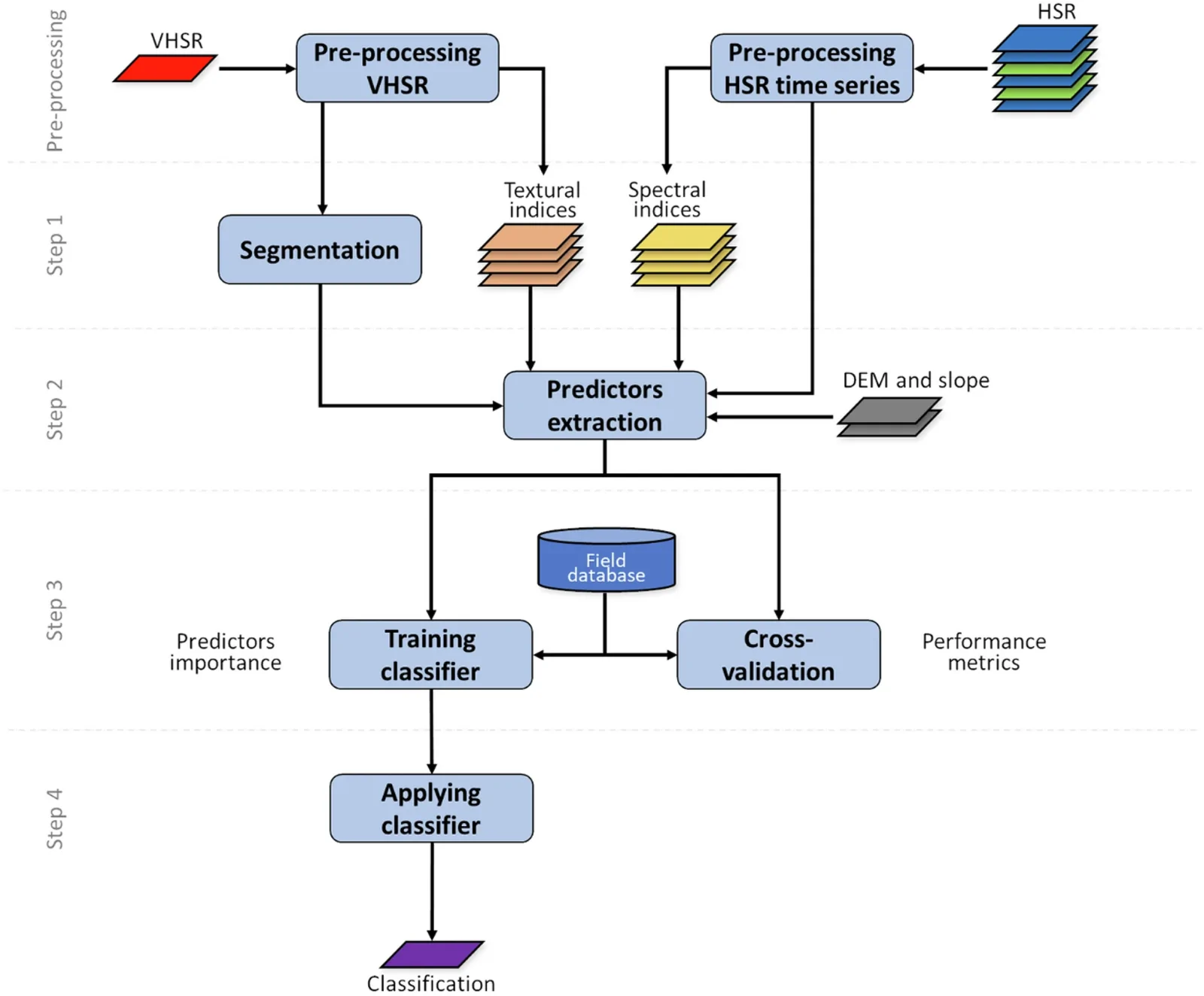
Overview of the main processing steps of the MORINGA processing chain [1], [16]: (1) VHSR image segmentation; (2) object-level feature extraction from multi-temporal Sentinel-2 and VHSR imagery; (3) Random Forest training and validation using the labeled polygon dataset; (4) LULC map production.

Fig. 4 presents examples of annual LULC maps produced for the OBSYDYA pilot sites at 1.5 m spatial resolution. The maps illustrate the spatial diversity of the agricultural landscapes represented in the reference dataset, including annual crops, perennial plantations, natural woody formations, shrubby savanna and non-agricultural surfaces.

**Fig. 4:**
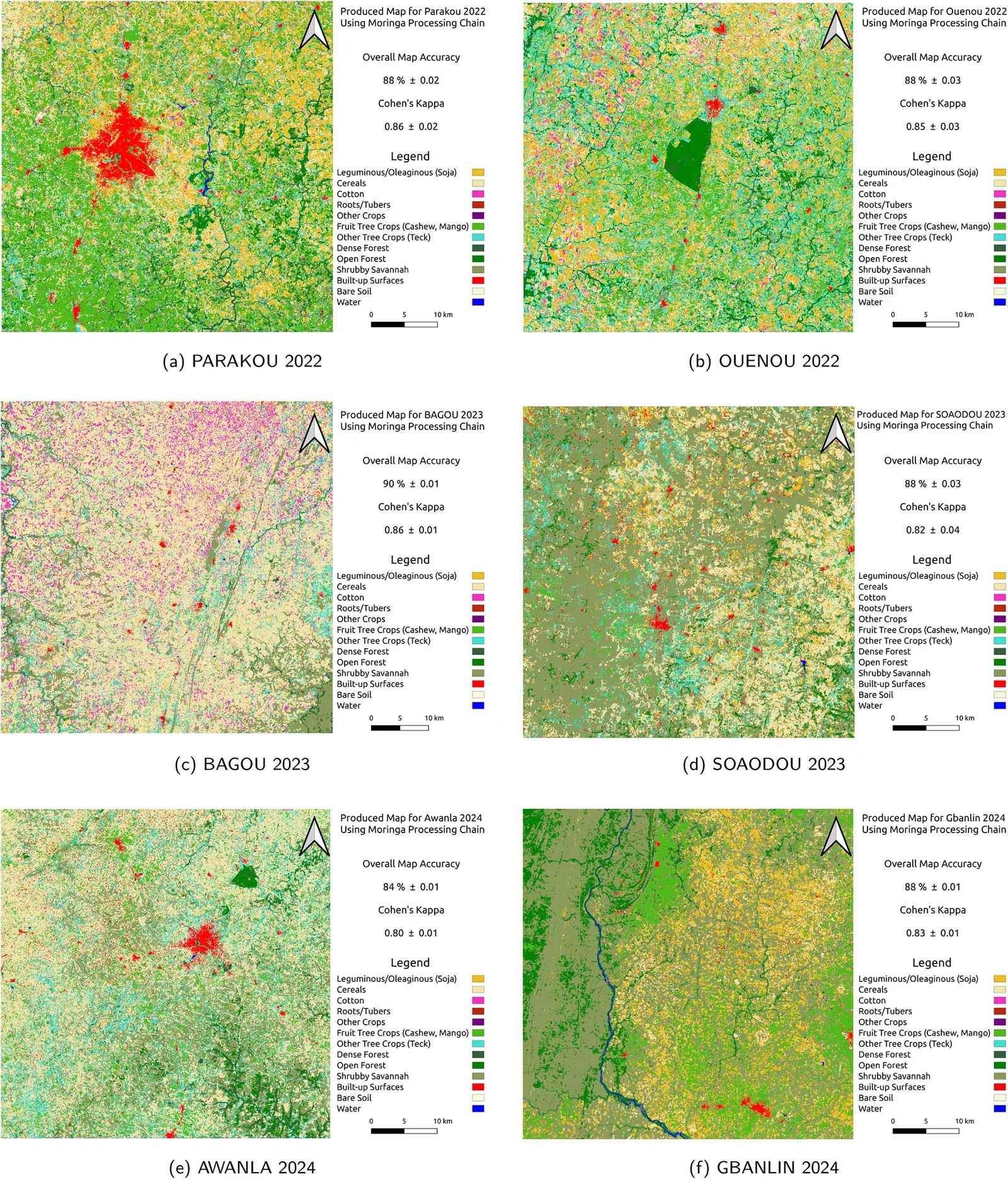
Example of an annual LULC map produced by the MORINGA processing chain [1] for OBSYDYA pilot sites. Colors correspond to land cover classes as defined in Table 1.

## Limitations

The temporal coverage of the dataset is uneven across sites: for annual crop types, three annual campaigns (2022–2024) are available for Parakou, Ouenou, and Bagou, while only two campaigns (2023–2024) are available for Soaodou, Awanla, and Gbanlin. This limits the interannual analysis that can be conducted for the less-covered sites. Furthermore, since crop rotations are frequent in this region, annotations from one year cannot be directly reused for another campaign, and each year’s reference data must be treated as independent. A last field campaign has been performed in November 2025, which will soon enable the update of this dataset with the addition of a further year on all the six sites.

The heterogeneity of farming practices in the study area, combined with the predominance of smallholder plots typically below 0.25 ha, results in a large proportion of small polygons potentially introducing mixed-pixel effects at class boundaries. In addition, the dataset exhibits a marked class imbalance reflecting the actual land cover distribution of the surveyed landscapes: perennial classes such as Fruit Trees and Shrubby Savanna dominate in spatial extent at several sites, whereas classes such as Water, Bare Soil, Dense Forest, and Other Crops are considerably less represented in the reference data.

Finally, field surveys were conducted exclusively during October and November. While this period is favorable for identifying most annual crops at their advanced phenological stage, some land cover types such as early-season crops, wetland surfaces at peak extent, or certain fallow dynamics may be underrepresented or misclassified in the dataset.

## Funding

This work is supported by funds from the European Union (EU) via the OBSYDYA Project (DESIRA, #FOOD/2020/417-846).

## CRediT authorship contribution statement

**Sabi Bruno Bio Nikki Sarè:** Conceptualization, Investigation, Data curation, Methodology, Software, Writing – original draft. **Raffaele Gaetano:** Conceptualization, Investigation, Data curation, Methodology, Validation, Software, Writing – original draft. **Yvon-Carmen Hountondji:** Methodology, Data curation, Supervision, Funding acquisition, Project administration, Writing – review & editing. **Comlan Hervé Sossou:** Investigation, Data curation, Supervision, Funding acquisition, Project administration, Writing – review & editing. **Laurent Gazull:** Investigation, Data curation, Supervision, Funding acquisition, Project administration, Writing – review & editing. **Gérard Nounagnon Gouwakinnou:** Investigation, Data curation, Supervision, Visualization. **Nestor René Ahoyo Adjovi:** Investigation, Data curation, Supervision, Visualization, Project administration. **Simon Madec:** Software, Investigation, Data curation, Supervision, Visualization. **Agnès Bégué:** Investigation, Data curation, Supervision, Visualization. **Naboua Abdelkader Kouhoundji:** Investigation, Data curation, Visualization, Supervision. **Omoto Aurelle Christelle Sedegnan:** Investigation, Data curation, Visualization. **Mikhaïl Jean De Dieu Dotou Padonou:** Investigation, Data curation, Visualization. **Coômlan Charles Hounton:** Investigation, Data curation, Supervision. **Séverin Biaou:** Investigation, Data curation, Supervision. **Jaurès Kakpo:** Investigation. **Samirath Chabi Yoruba:** Investigation. **Nicanor Kouton:** Investigation. **Bénoît Adinafo Clément Bessan:** Visualization, Data curation. **Biaou Denis Olou:** Investigation, Data curation, Supervision. **Jean Yarou Koto:** Investigation, Visualization. **Amah Akodewou:** Investigation, Visualization, Data curation. **Benoit Mertens:** Investigation, Data curation, Supervision. **Antoine Denis:** Supervision, Visualization. **Bernard Tychon:** Supervision, Funding acquisition, Project administration. **Jonathan Peereman:** Investigation, Data curation. **Valentine Lebourgeois:** Investigation, Visualization. **Roberto Interdonato:** Supervision, Supervision, Methodology, Writing – review & editing. **Jean Huchon:** Visualization, Funding acquisition, Project administration.

## Footnotes

1 https://www.obsydya.org

2 https://inrab.bj/recherche_developpement

3 https://qfield.org
